# Parameterization of Boronates Using VFFDT and Paramfit for Molecular Dynamics Simulation

**DOI:** 10.3390/molecules25092196

**Published:** 2020-05-08

**Authors:** Barış Kurt, Hamdi Temel

**Affiliations:** 1 Institute of Science, Department of Chemistry, Dicle University, 21280 Diyarbakir, Turkey; 2 Faculty of Pharmacy, Department of Pharmaceutical Chemistry, Dicle University, 21280 Diyarbakir Turkey; htemel@dicle.edu.tr

**Keywords:** Amber, force field, parameterization, boron

## Abstract

Boric acid, borate esters, and hydroxy derivatives are biologically active molecules. Thus, performing molecular dynamics simulations of these molecules is vital in terms of drug design, but it is difficult to find directly generated Amber parameters based on an ab initio method for these kinds of molecules in the literature. In this study, Amber parameters for such molecules containing boron were generated based on ab initio calculations using the paramfit program, which applies a combination of genetic and simplex algorithms, and the Visual Force Field Derivation Toolkit (VFFDT) program containing the Seminario method. The minimized structure, after obtaining novel parameters and using the sander program, was compared with the experimental crystallographic structures, and it was observed that the root-mean-square deviation (RMSD) value between the experimental structure and minimized structure agreed reasonably well. In addition, the molecule was heated, and the molecular dynamics simulation was successfully obtained with the novel parameters.

## 1. Introduction

Boron compounds, which are known to be biologically active, are strong Lewis acids and can easily form coordinate covalent bonds with nucleophiles due to their empty *p* orbitals. In particular, boric acid, borate esters, and hydroxy derivatives are important for drug design because of their high stability and low toxicity under physiological conditions, and this design requires an efficient and inexpensive theoretical tool such as molecular dynamics simulations [1].

Performing a molecular dynamics simulation requires force-field parameters related to the molecule. Although the generation of these parameters varies slightly according to the program used, it is essential to generate non-bond, bond, angle, and dihedral parameters. In the literature, multiple ways of obtaining bond and angle parameters were shown. In the production of the General Amber Force Field (GAFF), angle and bond parameters were obtained from the same formula as Merck Molecular Force Field MMFF 94, while non-bond parameters were taken from the Assisted Model Building with Energy Refinement (Amber) force field [2]. Zhu et al., obtained parameters from the fitted molecular mechanics energy profile and potential energy surface (PES) scan [3]. Lin et al. used the Seminario method to obtain missing zinc parameters [4]. In the Seminario method, the force constants are calculated on the Hessian matrix; thus, obtained values are independent of the selected internal coordinates [5,6].

For Amber dihedral parameters, multiplicity and phase angle must be specified in the force-field file [7]. Therefore, the above-mentioned formulas and the Seminario method for angle and bonds cannot be used when calculating the dihedral parameters. Instead, the plot obtained from the rotational energy profile of dihedral is converted to the truncated Fourier series and then fit to molecular mechanics (MM) calculation. This can be done manually or by using an algorithm.

Since it takes a lot of time to do full force-field parameterization manually, various programs were written for this task. This article focuses on two of these programs. The first one is the VFFDT program, which implements the Seminario method and automatically assigns the GAFF atomic type for atoms [8]. The second program is paramfit for parameterization using a genetic and simplex algorithm combination [9]. In this study, the parameters required for molecular dynamics simulation of borate esters were generated and tested using these two programs.

## 2. Results

**Figure 1:**
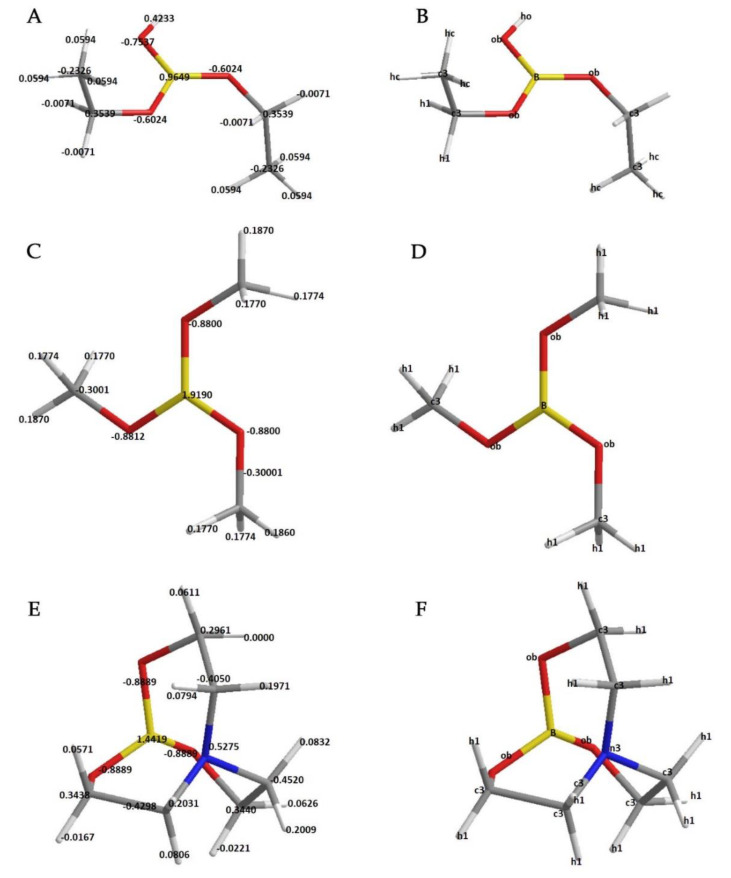
Electrostatic potential (ESP) charges (a.u.) and assigned atom types for molecules. (**A**,**C**,**E**) show ESP charges for diethoxyboronic acid, IVEKAW02, and TEAMBO04, respectively. (**B**,**D**,**F**) show assigned atom types in the same order.

Defined atom types and calculated quantum mechanical molecular electrostatic potentials (ESP charges) can be seen in Figure 1. All parameterization processes were accomplished according to these charges and atom types.

The bond and angle parameters generated as a result of the parameterization process can be seen in Table 1, and dihedral parameters can be seen in Table 2.

It should be noted that the h1–ob–B–ob and ob–B–ob–c3 dihedrals in Table 2 are the sum of more than one term; thus, periodicity of the first dihedral should be taken as negative [7]. There is no need for parameterization of all dihedrals related to the molecule. The wildcard terms in GAFF can also be used when necessary. For example, for B–ob–c3–c3 and B–ob–c3–h1 dihedrals, X–c–os–X in GAFF can be used; for the ob–c3–c3–h1 dihedrals, the terms for X–c–c–X in GAFF are used [2]. Improper dihedrals are described similarly [2,7], although defining improper dihedrals is not always a necessity. The suggested improper parameters in this study, which were calculated using paramfit, are given in Table 3.

**Figure 2:**
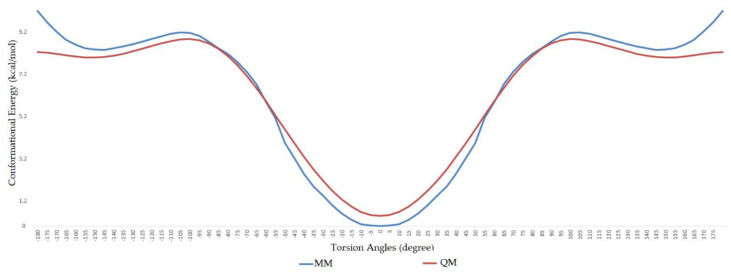
Comparison of ab initio and molecular mechanics calculations for ob–B–ob–c3 dihedral.

**Figure 3:**
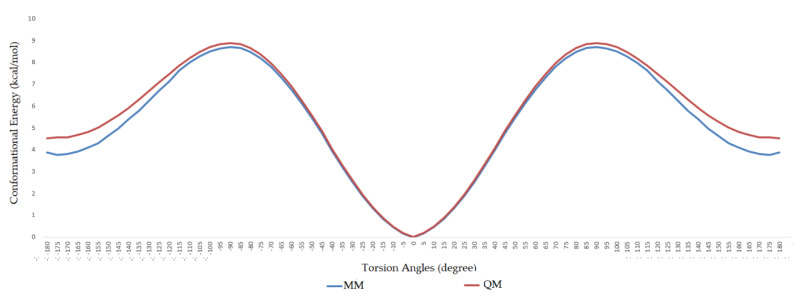
Comparison of ab initio and molecular mechanics calculations for h1–ob–B–ob dihedral.

During the dihedral parameterization, the c3–ob–B–ob dihedral was scanned in the range of −180° to +180° with an increase of 5°, and the energy profile difference between the ab initio calculation and molecular mechanics calculation can be seen in Figure 2. Similarly, the ob–B–ob–ho dihedral was scanned between −180° and +180°, and the difference between ab initio and MM calculation can be seen in Figure 3.

The minimized geometry of the molecule in vacuum with the sander program [7] was converted to a pdb file with the ambpdb command, and the minimized structure was compared with the crystallographic structure (see Table 4). While making this comparison, only the first three atoms that are directly adjacent to the boron atom were taken as the basis, and the others were ignored.

**Figure 4:**
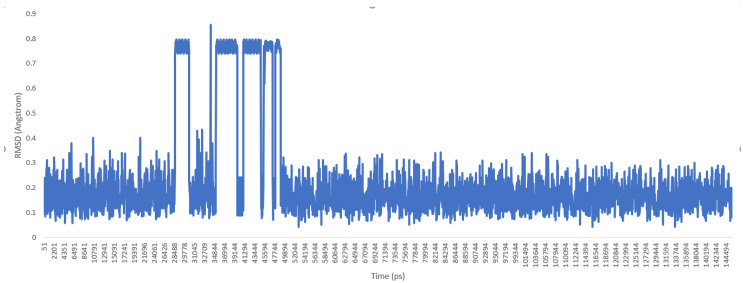
RMSD chart for IWEKAW02 molecular dynamics production (duration: 145 ns).

**Figure 5:**
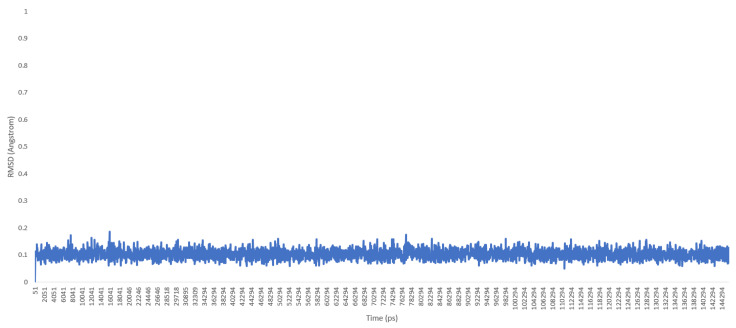
RMSD chart for TEAMBO04 molecular dynamics production (duration: 145 ns).

Then, the molecule was heated, and a molecular dynamics production was carried out. Trajectory files of the molecule during the production phase were loaded into the UCSF chimera program [10], and the root-mean-square deviation (RMSD) graphic was drawn (see Figure 4 and Figure 5)

## 3. Discussion

As a previously mentioned, the power of the molecular dynamics parameters is that it can reproduce experimental data. When the force-field studies in the literature were examined, the values of RMSD-AD (atomic displacement), RMSD-L (bond length), and RMSD-A (bond angle) for GAFF [2] increased to 0.992, 0.0477, and 4.12, respectively; the RMSD value increased to 43.8° for the MM2X out-of-plane angle [11]. If Table 3 is examined, it can be observed that RMSD-L and RMSD-A values did not exceed 0.212 and 0.039, respectively, which is in conformity with the literature [2,11]. Table 4 shows the calculated numbers, denoted inside the parentheses, where out-plane numbers are not included. It can be observed that these values did not exceed 3.381 and remained below 4.12. According to these results, our novel parameters are able to reproduce experimental data reasonably well, and they confirmed the reliability of bond and angle parameters by reproducing geometry very close to the X-ray structure. In addition, the reliability of the parameters produced by the Seminario method was already proven many times in the literature. It is also clear from Figure 2 and Figure 3 that the molecular mechanics potential energy surface (PES) scan for dihedrals satisfactorily reproduced the corresponding PES using the B3LYP/6,311G ++ (2*d*, 2*p*) basis set. Additionally, IWEKAW02 started to stabilize after 28 ns, whereas TEAMBO04 maintained its initial stable state (Figure 4 and Figure 5). Therefore, it can be suggested that the molecular dynamics production was obtained successfully with our novel parameters.

## 4. Materials and Methods

Parameter-deriving studies were performed according to the Amber force-field parameters, and the formula for the Amber potential energy function is as follows [2,7]:

(1) $$E_{total}=\sum_{bonds}K_{r}{\left(r-r_{eq}\right)}^{2}+\sum_{angles}K_{\theta }{\left(\theta -\theta_{eq}\right)}^{2}\;+\sum_{dihedrals}\frac{V_{n}}{2}x\left[1+cos\left(n\Phi -\gamma \right)\right]+\sum_{i<j}\left[\frac{A_{ij}}{R_{’j}^{12}}-\frac{B_{ij}}{R_{’j}^{6}}+\frac{q_{i}q_{j}}{\epsilon R_{’j}^{}}\right]$$

where $K_{r}$ and $K_{\theta }$ symbols are bond and angle force constants, respectively;,n is multiplicity, *γ* is phase angle, $r_{eq}$ is bond equilibrium distance, $\theta_{eq}$ is angle equilibrium, and A, B, and q are non-bond parameters [2,12,13].

Tafi et al. [14] used the values taken from the MM2 force field as non-bond parameters for boron and reported that the simulation was successfully performed for the Amber force field. The same parameters were previously used by Otkidach for the CHARMM force field and reported to be successful as well [15]. Therefore, in this study, these parameters were used as non-bond parameters for boron and accepted as 𝛜 = 0.034 kcal/mol and r = 1.98 Å [10,11].

Molecular optimization, single-point energy calculation, and vibrational data calculation were carried out with the B3LYP/6,311G ++ (2*d*, 2*p*) basis set using the GAMESS-US software [16] based on the diethoxyborinic acid molecule in Figure 1A and B; then, the Seminario method was applied using the VFFDT software based on the obtained vibrational data file, and the angle and bond parameters were generated [8]. A dihedral PES scan was also performed using the psi4 program with the same basis set [17], and the fitting protocol was applied with the paramfit program in the Amber Tools program package [7,9]. ESP charge calculation was carried out with the HF/6-31G* basis set using psi4 and multiwfn software [6,7,17].

Validating the accuracy of the novel parameters is crucial for molecular mechanics. The accuracy of a parameter is related to its ability to reproduce experimental data [2]. Thus, in order to validate the generated parameters in this study, minimization was performed using sander, and the difference between the minimized geometry and the crystallographic structure of the molecule was investigated. Crystallographic structure mol2 files for TEAMBO04 and IVEKAW02 taken from The Cambridge Structural Database [18].

Angle and dihedral parameters were calculated based on optimized diethoxyborinic acid molecule as mentioned above. The *sp*^2^ hybridized Boron atom was defined as B, and the oxygen atom directly connected to this atom was defined as ob. For the ob novel atom type, o parameters in the GAFF were used as non-bond parameters (r =1.6612 Å, 𝛜 =0.2100 kcal/mol) [2,19]. For other atoms, the GAFF atom type was used [2]. For assigning GAFF atom types, the sybyl mol2 file was converted into GAFF atom type by using the antechamber program with -dr no and -j5 flags [2,7,20]; then, oxygen atoms connected to boron were replaced with ob. With the parmchk2 program [7], it was determined which parameters were required; then, after these parameters were placed in the force-field modification (*.frcmod) file, the molecule was minimized in the vacuum. Next, the molecule was heated up to 325 K at 100° steps, and the molecular dynamics simulation was obtained at 325 K for 145 ns [7]. Molecular geometry was assumed to correspond to the solid-state structure, which may not be the case when structures in solution are considered.

The SHAKE algorithm was used throughout in both heating and simulation runs using the ntc = 2 flag. In Amber, ntc = 2 means that only the hydrogen bond energy goes to zero and other bonds between heavy atoms still have energy [7]. The Berendsen thermostat was used for temperature control (ntt = 1), and the time constant for temperature coupling was set to 0.5 ps (tautp = 0.5). The time step was 2,500,000 × 0.002 ps (nstlim = 2,500,000, dt = 0.002) for each 5-ns file. The cut-off nonbonded interaction was specified according to a value of 999 Å (cut = 999) [7].

It is essential to prevent the effects of atoms outside the dihedral during scanning in order to obtain a seamless molecular mechanics scan graphic. For this purpose, all the coordinates for each step of the scan were extracted from the psi4 output file, using the awk and sed commands under wsl Ubuntu OS, and these coordinates were converted into mol2 files by using the pymol [21] molecular editing program. Later, ESP charges were added to mol2 files, and mol2 files with ESP charge were converted to GAFF atom type using the "at gaff -dr no flags of the antechamber program [20]. Oxygen atoms associated with the boron atom were changed to ob. Then, the coordinate and prmtop files for each mol2 file were obtained using the tleap program and frcmod file containing our novel parameters (the tleap impose command was unable to turn dihedrals because of the boron atom) [7]. These mentioned files were used in the paramfit program, and the MM scan was generated based on the same geometries of the quantum mechanics (QM) scan.

## 5. Conclusions

As a result, the Amber force-field parameters for boronic acids and/or boronates were successfully generated and tested. It was observed that the ability of the produced parameters to reproduce experimental or quantum mechanics data remains within the limits specified in the literature in terms of RMSD. The molecule was also heated, and a molecular dynamics production was successfully accomplished. These parameters can be used in further molecular dynamics simulations for boron compounds with similar dihedrals and angles.

## Figures and Tables

**Table 1:** Generated bond and angle Amber force-field parameters based on optimized diethoxyborinic acid using the VFFDT program containing the Seminario method.

**Bond**	**K_r_ (kcal(mol∙Å^2^)^−1^**	**r_eq_ (Å)**
h1–ob	546.139	0.970
ob–B	350.221	1.372
ob–c3	219.866	1.435
**Angle**	**K_θ_ (kcal/(mol∙radian^2^)**	**θ_eq_ (°)**
h1–ob–B	50.184	110.55
ob–B –ob	104.357	120.00
B –ob–c3	110.735	121.35
ob–c3–c3	114.01	111.44
ob–c3–h1	77.700	107.94

**Table 2:** Generated dihedral Amber parameters. h1–ob–B–ob and ob–B–ob–c3 dihedrals were generated from ab initio calculation and paramfit program. For other dihedrals, General Amber Force Field (GAFF) wildcard parameters were used [2].

*Dihedral*	Divider	Vn (kcal/mol)	*γ*	n	
h1–ob–B –ob	1	2.350	0.000	−1.000	
h1–ob–B –ob	1	1.654	0.000	2.000	
ob–B –ob–c3	1	1.980	180.000	−1.000	
ob–B –ob–c3	1	1.472	180.000	2.000	
B –ob–c3–c3	2	5.400	180.000	2.000	same as GAFF X –c–os–X [2]
B –ob–c3–h1	1	5.400	180.000	2.000	same as GAFF X –c–os–X [2]
ob–c3–c3–h1	1	0.300	180.000	2.000	same as GAFF X –c–c–X [2]

**Table 3:** Suggested improper dihedrals.

*Improper*	Vn (kcal/mol)	*γ*	n
ob–ob–B–ob	40.5	180.0	2.0

**Table 4:** Root-mean-square deviation (RMSD) between crystallographic structure and minimized geometry. RMSD-AD is the RMSD of atomic displacement, RMSD-L is the RMSD of bond length, and RMSD-A is the RMSD of bond angle. The numbers in the parentheses represent the out-of-plane angles not included in RMSD-A.

Molecule	RMSD-AD	RMSD-L	RMSD-A
IVEKAW02	0.206	0.0468	2.179 (1.442)
TEAMBO04	0.212	0.0765	5.976 (3.381)
Average	0.209	0.06165	4.077 (2.411)
